# Domains of Attraction of Multivariate Extreme Value Distributions

**DOI:** 10.6028/jres.099.053

**Published:** 1994

**Authors:** Rinya Takahashi

**Affiliations:** Kobe University of Mercantile Marine, Kobe, Hyogo 658, Japan

**Keywords:** asymptotic joint distribution, dependence function, multivariate extreme statistics

## Abstract

Some necessary and sufficient conditions for domains of attraction of multivariate extreme value distributions are shown by using dependence functions. The joint asymptotic distribution of multivariate extreme statistics is also shown.

## 1. Introduction

Multivariate extreme value distributions have been studied by many authors, and their contributions are summarized by Galambos [1] and Resnick [2]. The purpose of this paper is to obtain some necessary and sufficient conditions for domains of attraction of the multivariate extreme value distributions. The joint asymptotic distribution of multivariate extreme statistics is also obtained. To study multivariate extreme value distributions and their domains of attraction, Sibuya [3] introduces the notion of a dependence function which is also used by Galambos [1]. A dependence function or copula is a useful notion to construct a family of joint distributions.

In this paper, basic arithmetical operations arc always meant componentwise (see Galambos [1], Chapt. 5).

Let (*X*_1_*_j_*,*X*_2_*_j_*,…,*X_kj_*),*j*=1,2,…,*n*, be a sample of size *n*, of a *k*-dimensional random vector with a distribution function *F*(*x*), The *i*-dimensional distribution function of the components *X_j_*_1_,*X_j_*_1_,…,*X_j_*_1_ will be denoted *F_j_*_1_*_j_*_2_*_−j_*_1_(*x_j_*_1_,*_:_x_j_*_2,_…,*x_j_*_1_)*−F_j_*_(r)_(*x_j_*_(i)_). We shall also use the notation ${\bar{F}}_{j1\cdots ji}\left(x_{j1},\ldots ,x_{ji}\right)={\bar{F}}_{j\left(1\right)}\left(x_{j\left(i\right)}\right)=P\left(X_{j1}>x_{j1},\ldots ,X_{j1}>x_{ji}\right)$. For *k−*1 and *_P_*∈(0,1), let *F*^−1^ (*p*)=inf {*x*:*F* (*x*)≥*P*}.

Let *Z_n_*.=(*Z*_1_*_n_*,…,*Z_kn_*), where Z*_in_*=max{*X_i_*_l_,…,*X_in_*}, i=1, 2,…*k*, and let us call *Z_n_* a multivariate extreme statistic.

If there exist *a_n_*>0, *b_n_*∈*R^k^*, *n*=1, 2,…(*a_n_*>0 means *a_in_*>0, *i*=l ,…*k*) such that (*Z_n_−b_n_*)/*a_n_* converges in distribution to a random vector *U* with a nondegenerate distribution *H* (i.e., all univariate marginals of *H* are nonde-gencrate), then *F* is said to be in the domain of attraction of *H*, *F*∈*D*(*H*) by symbol, and *H* is said to be a multivariate extreme value distribution. The convergence in distribution is equivalent to the condition

$$\underset{n\to \infty }{\lim}\;F^{n}(a_{n}x+b_{n})=H(x)$$ (1)

for ali *x*, because multivariate extreme value distributions are continuous.

We shall need the following lemma to prove a proposition in Sec. 2.

**Lemma 1.1**Equation (1)*is equivalent to*

$$\underset{n\to \infty }{\lim}n\left[1-F\left(a_{n}x+b_{n}\right)\right]=-\log\;H(x)$$

*for all x such that* 0*<H(x)*<I. (See Marshall and Olkin [4].)

## 2. Domains of Attraction

For any *k*-dimensional distribution *F*,

$$D_{F}(y)=F\left(F_{1}^{-1}\left(y_{1}\right),\ldots ,F_{k}^{-1}\left(yk\right)\right),y=\left(y_{1},\ldots ,y_{k}\right)\in {\left(0,1\right)}^{k}$$

is called the dependence function of *F*. In this section, we derive necessary and sufficient conditions for domains of attraction in terms of the dependence function.

**Proposition 2.1***Let F be a k-dimensional distribution and let H be a multivariate extreme value distribution with univariate marginals H_i_, i*= l,… *k Then the following statements are equivalent:*

1. *F*∈*D*(*H*).
2. *F_i_*∈*D* (*H*_1_), *i*=1,…,*k*, *and*
  $$\underset{s\to \propto }{\lim}s\left[1-D_{F}(y^{1/s})\right]=-\log D_{H}(y)for\;all\;y\in {\left(0,1\right)}^{k}.$$
3. *F_i_*∈(*H_i_*) *i*=1,…,*k*, *and*
  $$\underset{x↑1}{\lim}\frac{1-D_{F}(y^{1-x})}{1-x}=-\log D_{H}(y)for\;all\;y\in {\left(0,1\right)}^{k}.$$
4. *F_i_*∈*D*(*H_i_*), *i*=1,…,*k*, *and*
  $$\underset{y↓0}{\lim}\frac{1-D_{F}(y^{r})}{1-D_{H}(y^{r})}=1for\;all\;y\in {\left(0,1\right)}^{k}.$$

**Proof**, The proof is straightforward from Lemma 1.1, Theorem 5.2.3 and Lemma 5.4.1 of Galambos [1]. □

**Proposition 2.2***Let F be a k-dimensional distribution and let H be a multivariate extreme value distribution with univariate marginals H_i_, i*=1,…,*k*.

- *F*∈*D* (*H*) *if and only if F_i_*∈(*H_i_*), *i*= 1,…,*k, and the functions*
  $$d_{J(i)}(y_{J(i)})=\underset{n\to \infty }{\lim}n{\bar{D}}_{F_{J}(i)}({\left(y_{J(i)}\right)}^{1/n})$$
  *for each fixed vector J*(*i*)(*i*>1) *and for all y*∈(0,1)^1^ *are finite, and the function*
  $$D_{H}(y;r)=y_{1}\ldots y_{k}\exp\{\sum_{i-2}^{k}{\left(-1\right)}^{i}\sum_{1<j|<-<j_{i}\leq k}d_{j(i)}(y_{f(i)})\}$$
  *is a dependence function of H*.
- *The following inequalities hold*.
  $$D_{H}(y;2r+1)\leq D_{H}(y)\leq D_{H}(y;2r).$$
  *for a nonnegative integer r, where*
  $$D_{H}(y;r)=y_{1}\cdots y_{k}\exp\left\{\sum_{i-2}^{r}{\left(-1\right)}^{j}\sum_{1\leq j_{1}<\cdots <j_{i}\leq k}d_{j(i)}(y_{j(i)})\right\}$$
  *and D_n_*(*y;r*) *is a dependence function of a multivariate extreme value distribution*.

**Proof.** It is easily seen that for all *s*>0,

$$sd_{j(i)}({\left(y_{j(i)}\right)}^{1/s})=d_{f(i)}(y_{j(i)}).$$

From Theorems 5.3.1 and 5.2.4 of Galambos [1], we have the result. □

**Example 2.1** (See Examples 5.2.2 and 5.2.3 of Galambos [1].) For a Mardia’s distribution

$$\begin{array}{c} F\left(x_{1},x_{2}\right)=1-e^{-x_{1}}-e^{-x_{2}}+{\left(e^{x_{1}}+e^{x_{2}}-1\right)}^{-1},\\ D_{F}\left(y_{1},y_{2}\right)=y_{1}+y_{2}-1+{\left[\frac{1}{1-y_{1}}+\frac{1}{1-y_{2}}-1\right]}^{-1}, \end{array}$$

and

$$\begin{array}{c} n{\bar{D}}_{F}\left(y_{1}^{1/n},y_{2}^{1/n}\right)=n{\left[\frac{1}{1-y_{1}^{1/n}}+\frac{1}{1-y_{2}^{1/n}}-1\right]}^{-1}\\ \to -\frac{\left(\log\;y_{1})(\log\;y_{2}\right)}{\log\;y_{1}+\log\;y_{2}},\;as\;n\to \infty . \end{array}$$

Thus, by Proposition 2.2 we have *F* ∈ *D*(*H*), where

$$\begin{array}{c} D_{H}\left(y_{1},y_{2}\right)=y_{1},y_{2}\exp\left[-\frac{\left(\log\;y_{1})(\log\;y_{2}\right)}{\log\;y_{1}+\log\;y_{2}}\right],\\ H\left(x_{1},x_{2}\right)-\Lambda \left(x_{1}\right)\Lambda \left(x_{2}\right)\exp\left\{1/\left(e^{x_{1}}+e^{x_{2}}\right)\right\}, \end{array}$$

and Λ(*x*)=exp(−e^−^*^x^*).

**Proposition 2.3***Let F and G be k-dimensional distri-butions and let H be a multivariate extreme value distribution*.

1. *If*, *G*∈*D*(*H*), *then*
  $$\underset{y↓0}{\lim}\frac{1-D_{F}(y^{1})}{1-D_{G}(y^{1})}=1for\;all\;y\in {\left(0,1\right)}^{k}.$$
2. *If F∈D* (*H*), *G*_1_*∈D*(*H*_1_) *i−*1,…,*k. and*
  $$\underset{y↑1}{\lim}\frac{1-D_{F}(y)}{1-D_{G}(y)}=1,\;where\;1=(1,\ldots ,1),$$
  *then G*∈*D*(*H*).

## 3. Marginally Independent or Perfect Dependent Multivariate Extreme Value Distributions

Let *H* be a multivariate extreme value distribution with univariate marginals *H_i_, i=*1,…,*k*, Let H_*_;(*x*)=*H*_1_(*x*_1_)…*H_k_*(*x_k_*);and H^*^(*x*)=min{*H_t_*(*x_t_*), *i=1* ,…, *k*}, then it holds

$$H\cdot (x)\leq H(x)\leq H^{\prime }(x)$$

for all *x*∈*K^k^*. Both bounds, H_*_ and H^*^, are multivariate extreme value distributions. Characterizations of these distributions are obtained by Takahashi [5].

In the bivariate case Sibuya [3] obtains necessary and sufficient conditions for *F*∈*D*(H_*_) and *F*∈*D*(H^*^). In this section we generalize his results.

**Proposition 3.1***Let F be a k-dimensional distribution and let H_i_ be a univariate extreme value distribution, i*=1,…,*k*. *Then the following statements are equivalent:*

1. *F*∈*D*(H_*_).
2. *F_i_*∈*D* (*H_i_*), *i*=1,…,*k and there exists y*∈(0,1)^k^ *such that*
  $$\underset{n\to \infty }{\lim}{\left(D_{F}(y^{1/n})\right)}^{n}=y_{1}y_{2}\cdots y_{k}.$$
3. *F_i_*∈*D*(*H_i_*), *i*=1,…,*k*, *and*
  $$\underset{y↑1}{\lim}\frac{1-D_{F}(y1)}{1-y}=k.$$
4. F*_i_*∈*D*(*H_i_*). i=1,…,*k*, *and*
  $$\underset{y↑1}{\lim}\frac{1-D_{F}(y1)}{1-y^{k}}=1.$$

**Proof.** The proof is straightforward from Theorems 2.2 and 4.1 and Corollary 2.4 of Takahashi [6]. □

**Remark.** If *k*=2, we have the same result as Proposition 3.1 by Corollary 2.2 of Takahashi [6].

**Example 3.1** (See Example 5.2.3 of Galambos [1].) For the Morgenstern distribution

$$\begin{array}{c} F\left(x_{1},x_{2}\right)=1-e^{-x_{1}}-e^{-x_{2}}+e^{x_{1}\;x_{2}}\left[1+\alpha \left(1-e^{-x_{1}}\right)\left(1-e^{-x_{2}}\right)\;\right],\\ D_{F}\left(y_{1},y_{2}\right)=y_{1}\cdot y_{2}\left[1+\alpha \left(1-y_{1}\right)\left(1-y_{2}\right)\right]. \end{array}$$

where −1≤α≤1, and

$$\underset{y↑1}{\lim}\frac{1-D_{F}(y\cdot y)}{1-y^{2}}=1.$$

By Proposition 3.1 4) we have *F*∈*D* (*H*.), where H_*_(·,·)=*Λ*(·)*Λ*(·).

**Proposition 3.2***Let F be a k-dimensional distribution and let H_i_ be a univariate extreme value distribution, i*=1,…,*k. Then the following statements are equivalent:*

1. *F*∈*D*(H^*^).
2. *F_i_*∈*D*(*H_i_*), *i*=1,…,*k*, *and there exists y*∈(0,1) *such that*
  $$\underset{n\to \infty }{\lim}{\left(D_{F}(y^{1/n}1)\right)}^{n}=y.$$
3. *F_i_*∈*D* (*H_i_*), *i*=1,…,*k. and*
  $$\underset{y↑1}{\lim}\frac{1-D_{F}(y1)}{1-y}=1.$$

**Proof.** The proof is straightforward from Theorem 3.1 and Corollary 3.1 of Takahashi [6]. □

## 4. Joint Asymptotic Distribution of the Multivariate Extreme Statistics

In this section, we show the joint asymptotic distribution of several multivariate extreme statistics along the arguments in Sec. 2.3 of Leadbetter et al. [7]. For simplicity we shall consider the bivariate case.

Let (*X*_1_*Y*_1_),…,(*X_n_*,*Y_n_*) be a sequence of independent random vectors with common distribution *F*. The order statistics of the components will be denoted by

X1:n≤X2:n≤⋯≤Xn:n;andY1:n≤Y2:n≤⋯≤Yn:n.

For *i*−0,1,…,*r*=1, define

$$Z_{n-1}=(X_{n-1:n},Y_{n-1:n})$$

and let us call Z*_n i_* an (*i*+1)-th multivariate extreme statistic.

**Proposition 4.1***Suppose that*

$$P\{(Z_{n}-b_{n})/a_{n}\leq x\}\overset{w}{\to }H(x)$$

for some nondegenerate distribution H_*_ Then, for x_1_=(*x*_1_,*y*_1_)>*x*_2_=(*x*_2_,*y*_2_).

$$P\left\{\left(Z_{n}-b_{n}\right)/a_{n}\leq x_{1},\left(Z_{n-1}-b_{n}\right)/a_{n}\leq x_{2}\right\}\overset{w}{\to }H_{1}\left(x_{1},x_{2}\right)$$

*where*

$$\begin{array}{c} H_{1}\left(x_{1},x_{2}\right)=H\left(x_{2}\right)\{1+\log\;H\left(x_{1}\right)-\log\;H\left(x_{2}\right)\\ +\left[\log\;H_{1}\left(x_{1}\right)-\log\;H_{1}\left(x_{2}\right)+\left(h\left(x_{1},y_{2}\right)-h\left(x_{2}\right)\right)\right]\\ \times \left[\log\;H_{2}\left(y_{1}\right)-\log\;H_{2}\left(y_{2}\right)+\left(h\left(x_{2},y_{1}\right)-h\left(x_{2}\right)\right)\right]\} \end{array}$$

*and* $h\left(x\right)=\lim_{n\to \infty }\bar{F}\left(a_{n}x+b_{n}\right)$.

**Proof.** Define

$$\begin{array}{l} S_{0}^{n}=\#\left\{j|X_{j}>a_{1n}x_{1}+b_{1n}\;or\;Y_{j}>a_{2n}y_{1}+b_{2n},j=1,2\ldots ,n.\right\},\\ S_{1}^{n}=\#\{j|a_{1n}x_{2}+b_{1n}<X_{j}\leq a_{1n}x_{1}+b_{1n}\;\mathrm{and}\\ \;Y_{j}\leq a_{2n}y_{2}+b_{2n},j=1,2,\ldots ,n.\},\\ S_{2}^{n}=\#\{j|X_{j}\leq a_{1n}x_{2}+b_{1n}\;\mathrm{and}\\ \;a_{2n}y_{2}+b_{2n}<Y_{j}\leq a_{2n}y_{1}+b_{2n},j=1,2,\ldots ,n.\},\\ S_{12}^{n}=\#\left\{j|a_{n}x_{2}+b_{n}<\left(X_{j},Y_{j}\right)\leq a_{n}x_{1}+b_{n},j=1,2,\ldots ,n.\right\} \end{array}$$

then, we have

$$\begin{array}{c} P\left\{\left(Z_{n}-b_{n}\right)/a_{n}\leq x_{1},\left(Z_{n-1}-b_{n}\right)/a_{n}\leq x_{2}\right\}\\ =P\left\{S_{0}^{n}=0,S_{12}^{n}=0,S_{1}^{n}\leq 1,S_{2}^{n}\leq 1\right\}\\ +P\left\{S_{0}^{n}=0,S_{12}^{n}=1,S_{1}^{n}=0,S_{2}^{n}=0\right\}. \end{array}$$

On the other hand, by using Theorem 5.3.1 of Galambos [1], we can evaluate the asymptotic probabilities of the evens

$$\left\{S_{0}^{n}=i,S_{12}^{n}=j,S_{1}^{n}=k,S_{2}^{n}=m\right\}$$

for *i,j,k,m*=0,1,Thus we have the result. □

**Corollary 4.1***Suppose that*

$$P\left\{\left(Z_{n}-b_{n}\right)/a_{n}\leq x\right\}\overset{w}{\to }H\left(x\right)$$

*for some nondegenerate distribution H, Then, for fixed r*≥1 *and x*_1_>…*x_r_*

$$\begin{array}{l} |P\left\{\left(Z_{n}-b_{n}\right)/a_{n}\leq x_{1},\ldots ,\left(Z_{n-r+1}-b_{n}\right)/a_{n}\leq x,\right\}\\ -P\left\{\left(Z_{n}^{*}-\beta_{n}\right)/\alpha_{n}\leq x_{1},\ldots ,\left(Z_{n-r+1}^{*}-\beta_{n}\right)/\alpha_{n}\leq x_{r}\right\}|\to 0,\\ as\;n\to \infty , \end{array}$$

*where*$Z_{n-1}^{*}$is the (*i+*1)-*th multivariate extreme statistic from the distribution H*, *i*=0,…, *r−*1, *and H^n^*(α*_n_x+β_n_*)=*H* (*x*), *n*=1,2,…

**Example 4.1** Let *F* be the bivariate normal distribution with the correlation coefficient less than one. Then

$$\begin{array}{l} |P\left\{\left(Z_{n}-b_{n}\right)/a_{n}\leq x_{1},\ldots ,\left(Z_{n-r+1}-b_{n}\right)/a_{n}\leq x_{r}\right\}\\ -P\left\{(Z_{n}^{*}-\left(\log\;n\right)1\leq x_{1},\ldots ,(Z_{n-r+1}^{*}-\left(\log\;n\right)1\leq x_{r}\right\}|\to 0,\\ as\;n\to \infty , \end{array}$$

where $Z_{n-1}^{*}$ is the (*i*+1)-th multivariate extreme statistic from the bivariate exponential distribution whose marginals are equal to the standard exponential distribution and they are independent. For the univariate case, it is a well known result (see Weissman [8], Theorem 3).
